# Antinociceptive Activity of *Trichilia catigua* Hydroalcoholic Extract: New Evidence on Its Dopaminergic Effects

**DOI:** 10.1093/ecam/nep144

**Published:** 2011-04-14

**Authors:** Alice F. Viana, Izaque S. Maciel, Emerson M. Motta, Paulo C. Leal, Luiz Pianowski, Maria M. Campos, João B. Calixto

**Affiliations:** ^1^Department of Pharmacology, Universidade Federal de Santa Catarina, Campus Universitário, Trindade, Florianópolis, SC, Brazil; ^2^Faculty of Pharmacy, Pontifícia Universidade Católica do Rio Grande do Sul, Porto Alegre, RS, Brazil; ^3^Department of Chemistry, Universidade Federal de Santa Catarina, Campus Universitário, Trindade, Florianópolis, SC, Brazil; ^4^Pianowski and Pianowski Consulting, Brazil; ^5^Faculty of Dentistry, Pontifícia Universidade Católica do Rio Grande do Sul, Porto Alegre, RS, Brazil

## Abstract

*Trichilia catigua* is a native plant of Brazil; its barks are used by some local pharmaceutical companies to prepare tonic drinks, such as Catuama. The present study was addressed to evaluate the effects of *T. catigua* hydroalcoholic extract in mouse nociception behavioral models, and to evaluate the possible mechanisms involved in its actions. Male Swiss mice were submitted to hot-plate, writhing and von Frey tests, after oral treatment with *T*. *catigua* extract (200 mg kg^−1^, p.o.). The extract displayed antinociceptive effect in all three models. For characterization of the mechanisms involved in the antinociceptive action of the extract, the following pharmacological treatments were done: naloxone (2.5 mg kg^−1^, s.c.), SR141716A (10 mg kg^−1^, i.p.), SCH23390 (15 **μ**g kg^−1^, i.p.), sulpiride (50 mg kg^−1^, i.p.), prazosin (1 mg kg^−1^, i.p.), bicuculline (1 mg kg^−1^, i.p.) or dl-*p*-chlorophenylalanine methyl ester (PCPA, 100 mg kg^−1^, i.p.). In these experiments, the action of *T. catigua* extract was evaluated in the hot-plate test. The treatment with SCH23390 completely prevented the antinociceptive effect, while naloxone partially prevented it. The possible involvement of the dopaminergic system in the actions of *T. catigua* extract was substantiated by data showing the potentiation of apomorphine-induced hypothermia and by the prevention of haloperidol-induced catalepsy. In conclusion, the antinociceptive effects of *T. catigua* extract seem to be mainly associated with the activation of dopaminergic system and, to a lesser extent, through interaction with opioid pathway.

## 1. Introduction

*Trichilia catigua* A. Juss (Meliaceae) is a native plant that grows abundantly in several regions of Brazil, and it is popularly known as “catuaba” or “catigua” [1]. This plant is widely used in folk medicine as a tonic for the treatment of fatigue, stress, impotence and against deficits of memory, being also employed as a digestive and purgative substance. Some pharmaceutical and beverage industries in Brazil use the barks of this plant to prepare tonic drinks. Commercially available preparations containing *T. catigua* extracts often include the association of other plants, with known stimulant properties. In Brazil, the herbal product Catuama is made by the association of four hydroalcoholic extracts, namely *T. catigua* (28.23%), *Paullinia cupana* (40.31%), *Ptychopetalum olacoides* (28.23%) and *Zingiber officinalis* (3.26%). The product Catuama has been commercialized in Brazil for more than 20 years, and it is used as a remedy to relieve physical and mental fatigue, neuromuscular asthenia and weakness disorders.

Concerning its pharmacological actions, Catuama has been previously shown to display marked vasorelaxant actions in vascular preparations obtained from rats, guinea-pigs and rabbits [2], besides possessing long-lasting (up to 8 h) antinociceptive effects in various thermal (hot-plate and tail flick) and chemical (acetic acid, formalin and capsaicin) acute nociception models [3]. The authors have also evaluated the effects of extracts made from the plants present in the product Catuama, *T. catigua, P. cupana*, *P. olacoides* and *Z. officinalis*, and verified that *T. catigua* had the higher antinociceptive effect on the acetic acid-induced nociception [3]. More recently, our group has shown that Catuama also produces a prominent reduction of the mechanical hypersensitivity induced by LPS in rats [4]. Additional pre-clinical studies have shown a relaxant action for Catuama in corpus cavernosum strips from rabbits [5], this effect was correlated with the presence of *P. cupana* and *T. catigua* extracts. Catuama has also been found able to both revert and prevent ventricular fibrillation in the isolated rabbit heart, and *T. catigua* extract is probably the main agent responsible for these actions [6]. Of interest, our group has provided pharmacological and biochemical evidence on the potential antidepressant effects of the product Catuama and also for the *T. catigua* hydroalcoholic extract in rodents, by mechanisms primarily involving the activation of dopaminergic pathways [7, 8]. Following these lines of evidence, the present study was addressed to further evaluate the effects of the hydroalcoholic extract from *T. catigua* barks on the central nervous system (CNS), by using some behavioral pharmacological models in rodents. In addition, efforts have also been made to further investigate the possible mechanisms underlying these effects, by using experimental models of pain evaluation.

## 2. Methods

### 2.1. Drugs and Reagents

Diazepan, imipramine hydrochloride, dexamethasone, fluoxetine hydrochloride, haloperidol, indomethacin, naloxone, _DL_-*p*-chlorophenylalanine methyl ester (PCPA), prazosin, SCH23390, morphine, carrageenan (all from Sigma Chemical Co. St. Louis, MO, USA); sulpiride (DEG, São Paulo, SP, Brazil); dypirone, acetic acid (Merck AG, Darmstadt, Germany) SR 141716A (Sanofi Aventis, São Paulo, SP, Brazil). All the drugs used in this study, including the extract of *T. catigua*, were dissolved in phosphate buffered saline (PBS, Laborclin Ltd., Pinhais, PR, Brazil) to the desired concentration just before use. The selected doses were chosen according to pilot experiments and also on the basis of literature data [8, 9].

For the preparation of the extract, botanical material of *T. catigua* A. Juss (Meliaceae) was classified by Dr Gerdt Guenther Hatschback. A sample of the plant was deposited at the Municipal Botanical Museum of Curitiba, PR, Brazil (voucher number 65901). The barks of *T. catigua* were minced and extracted with ethanol-water in a proportion of 4 : 1 (w/v), and maintained at 60°C for 4 h. The solvent was fully evaporated and concentrated.

### 2.2. Animals

Swiss male mice (25–30 g; *N* = 8–10 per group) purchased from the Central Biotery of the Federal University of Pelotas (Brazil), kept in a room controlled for temperature (22  ±  2°C) and humidity (60%–80%), under a 12 : 12 h light-dark cycle (lights on at 6:00 am), were used. Animals were kept in groups of 10 per cage (height 16 cm, width 34 cm, length 49 cm). The mice were acclimatized to the laboratory for at least 1 h prior to experiments and were used only once in each test. For all behavioral tests, animals were visually and acoustically isolated during the experimental sessions. All experiments were carried out between 08:00 am and 5:00 pm. The Ethics Committee of the Pontifícia Universidade Católica do Rio Grande do Sul approved all the experimental procedures (protocol number 2304-07/1). All efforts were made to minimize the number and suffering of animals.

### 2.3. Hot-Plate Test

The hot-plate test was used to measure the response latencies according to the method described previously [10]. Briefly, the animals were placed on the hot-plate apparatus (54  ±  1°C) to measure the baseline responsiveness. Subsequently, different groups of mice were treated with the hydroalcoholic extract from *T. catigua* (200 mg kg^−1^, p.o.), the positive control drug morphine (4 mg kg^−1^, s.c.), or PBS solution (10 ml kg^−1^, p.o.). Separate groups of mice received Fraction 2 (200 mg kg^−1^, i.p.), Compound 2 or cinchonain IIB (20 and 40 mg kg^−1^, i.p.), each one isolated from *T. catigua* hydroalcoholic extract by column chromatography; the doses employed in our study were chosen based on pilot studies. The responsiveness of animals in the hot-plate apparatus was registered between 3 and 6 h after treatment; each time point was represented by one group of mice (*n* = 10 per group). The results of the time-course curve were used to determine the schedules of treatment for the next experiments. A latency period (cutoff) of 30 s was defined as complete analgesia.

In order to evaluate the neurotransmitter systems involved in the antinociceptive effects of *T. catigua* extract, mice were pretreated with naloxone (non-selective opioid receptor antagonist, 2.5 mg kg^−1^, s.c.); SCH23390 (15 *μ*g kg^−1^, i.p.) and sulpiride (50 mg kg^−1^, i.p.), selective dopamine D_1_ and D_2_ receptor antagonists, respectively; prazosin (*α*_1_-adrenergic receptor antagonist, 1 mg kg^−1^, i.p.), bicuculline (GABA_A_ receptor antagonist, 1 mg kg^−1^, i.p.), _DL_-*p*-chlorophenylalanine methyl ester (PCPA, serotonin synthesis inhibitor, 100 mg kg^−1^ i.p., 4 days, once a day); or SR141716A (cannabinoid receptor antagonist, 10 mg kg^−1^, i.p.). The drugs were injected immediately after evaluating the baseline responsiveness, and 10 min before the administration of *T. catigua* hydroalcoholic extract (200 mg kg^−1^, p.o.).

### 2.4. Measurement of Mechanical Hypersensitivity

The mechanical nociceptive threshold of the hindpaw was determined as described elsewhere [11]. Through a wire mesh floor of a chamber, a series of nine von Frey hair monofilaments (Stöelting, Wood Dale, IL, USA), calibrated to produce incremental forces of 0.02, 0.04, 0.07, 0.16, 0.4, 0.6, 1.0, 1.4 and 2.0 g, were applied to the middle of the plantar surface of the right hindpaw for a maximum of 8 s, or until the animal displayed a nocifensive response, consisting of paw lifting and/or shaking. Testing was initiated with the 0.6 g filament. In the absence of a clear paw withdrawal response, increasingly stronger filaments were presented consecutively, until one of them was found to elicit such a response. If the 0.6 g filament elicited a response, filaments with decreasing strength were presented until determination of the first one which failed to cause paw withdrawal. Data was collected using the up-down method [12] to calculate the 50% mechanical paw withdrawal threshold (in g). Significant decreases in the 50% paw withdrawal threshold were interpreted as indicative of mechanical allodynia. In our procedure, the basal response was registered 24 h before the test, and only animals responding to 0.6 g  ±  20% were selected. The animals were submitted to preventive and therapeutic treatment regimens. In preventive regimen, mice were treated with *T. catigua* extract (200 mg kg^−1^, p.o.), PBS as the negative control, or dexamethasone (1 mg kg^−1^, i.p.) as the positive control, followed by intraplantar (i.pl.) injection of 50 *μ*l of carrageenan (300 *μ*g) into the right hindpaw. For the therapeutic treatment, carrageenan (300 *μ*g/50 *μ*l/paw i.pl.) was injected into the right hindpaw, and 1 h later, mice received the *T. catigua* hydroalcoholic extract (200 mg kg^−1^, p.o.), PBS as the negative control, or indomethacin (5 mg kg^−1^, i.p.) and morphine (5 mg kg^−1^, i.p.), as the positive control drugs.

### 2.5. Abdominal Constrictions Induced by Acetic Acid

The abdominal constrictions were assessed according to procedures described previously [13]. They comprise a contraction of the abdominal muscle, together with a stretching of the hind limbs in response to an i.p. injection of acetic acid (0.8%). Mice were pretreated with the hydroalcoholic extract of *T. catigua* (200 mg kg^−1^, p.o.), 4 h before acetic acid injection. Control animals received a similar volume of PBS (10 ml kg^−1^, p.o.) or dypirone (150 mg kg^−1^, p.o.), 1 h before the application of acetic acid. After the injection of the algogenic agent, mice were individually placed into glass cylinders of 20 cm diameter, and the abdominal constrictions were counted cumulatively over a period of 15 min.

### 2.6. Apomorphine-Induced Hypothermia

For this test, the procedures used were similar to those described previously [14]. Temperature measurements were performed in a temperature-controlled room (22  ±  2°C), between 3:00 pm and 5:00 pm. The mouse colonic temperature was recorded using a commercially available thermometer (Pro-check), which was dipped in Vaseline and inserted *∼*0.5 cm into a gently hand-restrained mouse. After recording their initial colonic temperature (*t* = 0), different groups of mice received either *T. catigua* hydroalcoholic extract (200 mg kg^−1^, p.o.) or PBS (10 ml kg^−1^, p.o.). Following the appropriate intervals of treatment (4 h for the extract and 1 h for the PBS-treated animals); the animals received apomorphine (16 mg kg^−1^, s.c.) or PBS. Their colonic temperature was recorded 30 and 60 min after the last treatment.

### 2.7. Catalepsy

In order to evaluate the effects of *T. catigua* extract on the catalepsy induced by the antipsychotic haloperidol (a dopaminergic receptor antagonist); different groups of mice were pre-treated with PBS (10 ml kg^−1^, p.o.) or with *T. catigua* extract (200 mg kg^−1^, p.o.). Following the appropriate intervals of time (4 h for the extract; 1 h for the control groups), the animals were treated with haloperidol (4 mg kg^−1^, i.p.) or PBS. Catalepsy was measured 30 min after haloperidol injection, by placing the forepaws over a 0.5 cm diameter horizontal glass bar, supported 4 cm above the floor by two 8 × 8 cm pieces of metal. The time that animals remained with both paws on the bar was measured up to 90 s.

### 2.8. Statistical Analysis

Results are presented as the mean  ±  SEM of 8–10 animals for each experimental group. Statistical comparison of the data was performed by two-way analysis of variance (ANOVA) followed by Bonferroni's post-test, or one-way ANOVA followed by Newman-Keuls' test. *P*-values ≤ .05 were considered significant.

## 3. Results

### 3.1. Trichilia catigua Extract Displays Antinociceptive Actions on the Hot-Plate Test

**Figure 1:**
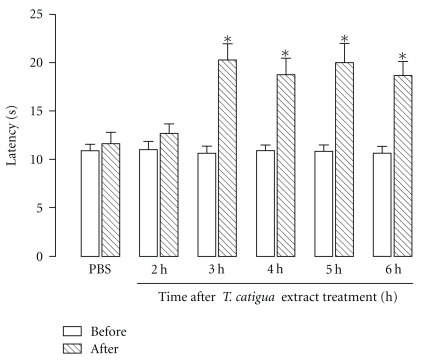
Time-course of the antinociceptive effect of *T. catigua* hydroalcoholic extract (200 mg kg^−1^, p.o) on the hot-plate test in mice. Results are presented as the mean  ±  SEM; *n* = 10–12 animals per group. *P < .05 significantly different from PBS-treated group (ANOVA followed by Newmann-Keuls post-hoc test).

The antinociceptive effects of *T. catigua* hydroalcoholic extract (200 mg kg^−1^, p.o) were initially investigated in an acute thermal model of nociception in mice. The time-course assessment demonstrated that *T. catigua* extract (200 mg kg^−1^) began to show antinociceptive action in the hot-plate paradigm only 3 h after its oral administration, an effect that lasted up to 6 h post-treatment (Figure 1). In contrast, the treatment of animals with the Fraction 2 (200 mg kg^−1^, i.p.), the Compound 2, or cinchonain IIB (20 and 40 mg kg^−1^, i.p.) did not produce any significant antinociceptive effect in this model (results not shown).

To evaluate some of the mechanisms involved in the antinociceptive effects of *T. catigua* extract in the hot-plate test, different groups of mice were pretreated with naloxone (2.5 mg kg^−1^, s.c.), SCH23390 (15 *μ*g kg^−1^, i.p.), sulpiride (50 mg kg^−1^, i.p.), prazosin (1 mg kg^−1^, i.p.), bicuculline (1 mg kg^−1^, i.p.), PCPA (100 mg kg^−1^, i.p.) or SR141716A (10 mg kg^−1^, i.p.). Only SCH23390 significantly prevent the antinociception caused by *T. catigua* extract (*P* = .04). The values of maximal possible effect (MPE) were calculated according to [15] and are compiled in Table 1.

### 3.2. Antinociceptive Effects of *T. catigua* Extract on Mechanical Hypersensitivity Induced by Carrageenan

**Figure 2:**
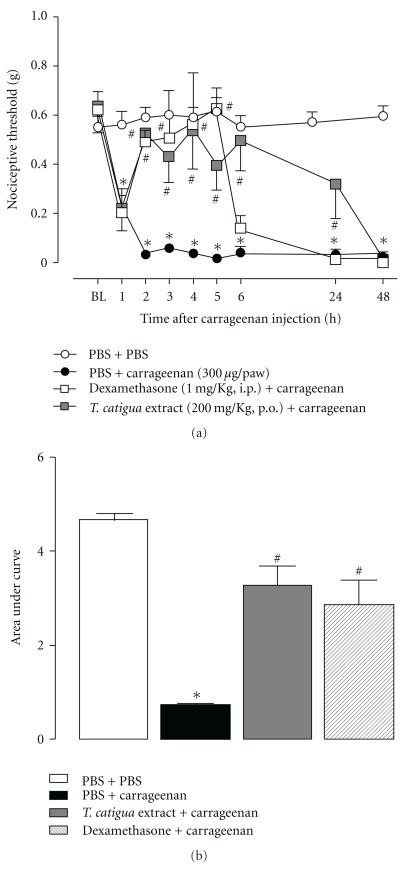
Effect of prophylactic treatment with *T. catigua* extract (200 mg kg^−1^, p.o.) and dexamethasone (1 mg kg^−1^, i.p.) on the mechanical hypersensitivity induced by carrageenan (300 *μ*g/50 *μ*l/paw, i.pl.) in mice. (a) Time-course of the anti-hypernociceptive effect; (b) AUC of the anti-hypernociceptive effect. Results represent the mean  ±  SEM; *n* = 6–8 animals per group. *P < .05 significantly different from PBS + PBS-treated mice; ^#^ *P* < .05 significantly different from PBS + carrageenan-treated mice (ANOVA followed by Newmann-Keuls post-hoc test).

**Figure 3:**
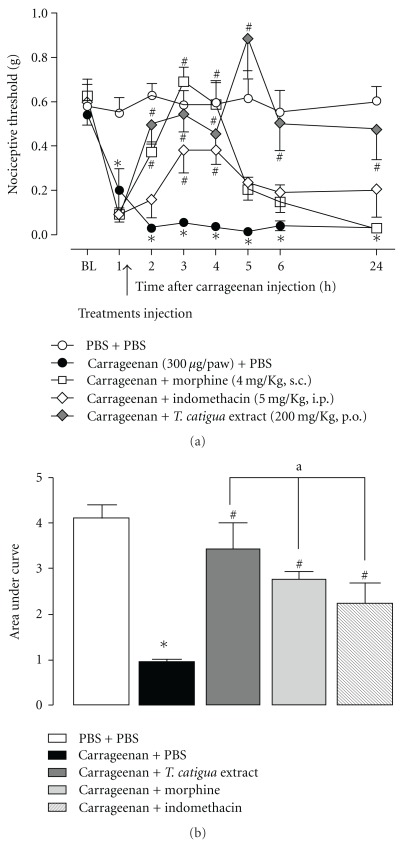
Effect therapeutic treatment with *T. catigua* extract (200 mg kg^−1^, p.o.), morphine (4 mg kg^−1^, s.c.) and indomethacin (5 mg kg^−1^, i.p.) on the mechanical hypersensitivity induced by carrageenan (300 *μ*g/50 *μ*l/paw, i.pl.) in mice. (a) Time-course of the anti-hypernociceptive effect; (b) AUC of the anti-hypernociceptive effect. Results represent the mean  ±  SEM; *n* = 6–8 animals per group. *P < .05 significantly different from PBS + PBS-treated mice; ^#^ *P* < .05 significantly different from PBS + carrageenan-treated mice; ^a^ *P* < .05 significantly different from morphine and indomethacin-treated mice (ANOVA followed by Newmann-Keuls post-hoc test).

When assessed in the mechanical hypersensitivity induced by carrageenan, *T. catigua* extract given orally, demonstrated both preventive and therapeutic antinociceptive actions. In both conditions, the antinociceptive actions of the extract lasted for up to 24 h, whereas those observed for dexamethasone, morphine and indomethacin (used as positive control drugs), lasted for 3–5 h (Figures 2 and 3). When the preventive schedule of administration was carried out, the area under the curve (AUC) revealed a marked antinociceptive effect for *T. catigua* extract (352  ±  38%), which was not significantly different from that obtained for dexamethasone (291  ±  41%) (Figure 2(b)). Furthermore, according to the calculation of the AUC, the therapeutic regimen of treatment with *T. catigua* extract displayed prominent antinociceptive effects (294  ±  47%), which were significantly higher in comparison to those observed for the positive control drugs, morphine (183  ±  20%) and indomethacin (131  ±  37%) (Figure 3(b)).

### 3.3. Treatment with *T. catigua* Extract Reduces Abdominal Constrictions Induced by Acetic Acid

**Figure 4:**
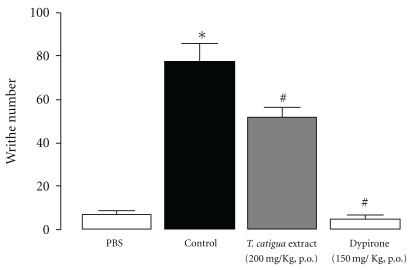
*T. catigua* extract (200 mg kg^−1^, p.o.) effect on the writhing test in mice. Results are in mean  ±  SEM; *n* = 10–12 animals per group. *P < .05 significantly different from PBS-treated mice; ^#^ *P* < .05 significantly different from control (PBS followed by acetic acid injection) treated mice (ANOVA followed by Newmann-Keuls post-hoc test).

In the writhing test, either the hydroalcoholic extract from *T. catigua* (200 mg kg^−1^, p.o., given 4 h prior) or dypirone (150 mg kg^−1^, p.o., 1 h) significantly reduced the number of writhes after acetic acid (0.8%) injection (*P* = .0002) (Figure 4). The obtained percentages of inhibition were 33  ±  6 and 94  ±  2% for the extract and dypirone, respectively.

### 3.4. Evidence for Involvement of Dopaminergic Pathways in the Actions of *T. catigua* Extract

**Figure 5:**
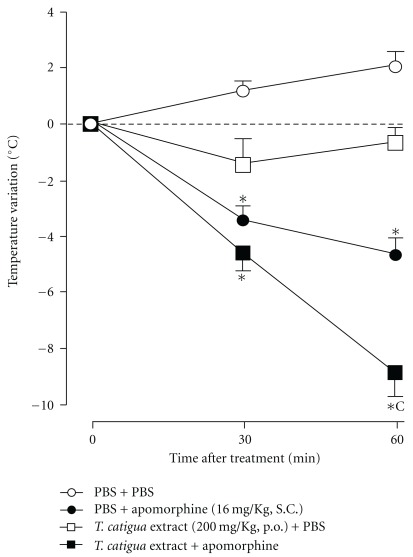
*T. catigua* extract (200 mg kg^−1^, p.o.) effect on apomorphine (16 mg kg^−1^, s.c.) induced hypothermia. Results are in mean  ±  SEM; *n* = 10–12 animals per group. *P < .05 significantly different from PBS-treated group; ^c^ *P* < .001 significantly different from apomorphine-treated group at the same time of measurement (two-way ANOVA followed by Newmann-Keuls post-hoc test).

*Trichilia catigua* extract did not display any direct dopaminergic-like activities, that is, it did not induce a reduction of body temperature or catalepsy *per se*, but it was able to significantly modify the effects of apomorphine and haloperidol. The hypothermia induced by apomorphine (16 mg kg^−1^, s.c.) was significantly enhanced by the pretreatment with *T. catigua* hydroalcoholic extract (200 mg kg^−1^, p.o.; *P* = .0001), given 4 h before apomorphine injection (Figure 5). In addition, the catalepsy induced by haloperidol was also significantly decreased by pretreating mice with *T. catigua* extract (*F*(5,45) = 14.74; *P* = .0003; Table 2).

## 4. Discussion

The evaluation of the effects of *T. catigua* hydroalcoholic extract on the CNS was initiated by Campos et al. [8]. The authors demonstrated that this plant extract displays antidepressant-like effects in the forced swimming test (FST), probably due to activation of the dopaminergic system and, to a lesser extent, the serotoninergic systems. In the present study we have provided additional evidence on the activities of *T. catigua* extract in the CNS, mainly through interaction with dopaminergic system.

We have initially assessed the effects of the extract in three classical nociception models in mice: the hot-plate, carrageenan-induced hypernociception and the writhing tests. The first is a widely accepted test related to the activation of central nociceptive pathways. The von Frey paradigm is usually employed to test analgesic/antiinflammatory substances, but the carrageenan hypersensitivity is also sensitive to CNS-acting substances. The last test is usually related to visceral pain, but since the parietal peritoneum receives somatic innervations, substances with central action are also effective in this model [16].

In our study, *T. catigua* extract produced marked, time-related and long-lasting antinociceptive effect in the hot-plate model. This result is in accordance with the previous one found by Vaz et al. [3], showing that Catuama effects started to decrease after 6 h. In this test, the antinociceptive activities of *T. catigua* are probably mediated by the dopaminergic system, and to a lesser extent, the opioid one. This conclusion is based on data showing that dopamine D_1_ receptor antagonist SCH23390 completely inhibited the extract antinociceptive effect in the hot-plate test, whilst the opioid antagonist naloxone prevented it only partially. Of relevance, it had been previously shown that Catuama prevented the hypersensitivity induced by Complete Freund's Adjuvant (CFA) and LPS in the von Frey test, and these effects were largely reversed by the non-selective dopamine receptor antagonist haloperidol [4]. The hypothesis that the antinociceptive effect of *T. catigua* extract is probably due to central analgesic rather than antiinflammatory activity is supported by the observation that Catuama, despite its anti-hypernociceptive effect against the i.pl. administration of LPS, was not effective in altering the production of the pro-inflammatory mediators IL-1*β*, TNF*α*, PGE_2_ or LTB_4_ [4]. Furthermore, *T. catigua* extract, at the same doses that it significantly reduced carrageenan-induced mechanical hypernociception, did not significantly alter the paw edema formation elicited by carrageenan injection (results not shown). However, the possible antiinflammatory effect of other extracts prepared from *T. catigua* cannot be completely ruled out. In this regard, Barbosa et al. [17] found that phospholipase A2 activity was totally inhibited by *T. catigua* extract.

Concerning the inhibitory descending pathways implicated in the control of pain, dopamine has not received as much attention as serotonin and norepinephrine [18]. The important role exerted by dopaminergic neurotransmission in modulating pain perception, opioid-induced antinociception and natural analgesia within supraspinal regions (including the basal ganglia, insula, anterior cingulate cortex, thalamus and periaqueductal gray) has only been demonstrated during the last couple of decades [19–21]. It is generally accepted that D_2_ receptor agonists elicit antinociception, while D_1_ receptor agonists induce pronociceptive effects [22, 23]. However, this separation remains as a matter for discussion. A great body of evidence suggests a close relationship between the opioid and dopaminergic systems [24–27]. In the striatum, neurones bearing D_1_ receptors also express the opioid peptide dynorphin, whereas cells containing D_2_-like receptors express enkephalin. There are studies showing that deletion of D_2_ receptors, by i.c.v. administration of an antisense probe directed against D_2_, enhances morphine antinociceptive actions over *μ* receptors, in a dose-dependent manner [28]. The involvement of D_1_ in the opioid analgesic effect is supported by some studies showing that periaquedutal gray dopaminergic pathways participate in supraspinal nociceptive responses after treatment with opiates [29].

Antidepressants, mainly mixed serotonin and noradrenaline reuptake inhibitors (tricyclics and venlafaxine), are widely prescribed for the treatment of chronic and neuropathic pain [30–33]. Nevertheless, the dopaminergic antidepressant, bupropion, has also shown antinociceptive properties in animal models [34]. The nature and underlying mechanisms of antidepressant analgesia is currently a matter of debate, but there is evidence showing that antidepressants may induce the release of endogenous opioids, and that this effect seems to be independent of their antidepressant effect [30, 35]. Besides that, some authors support the idea that the analgesic effects of antidepressants, such as nomifensine and dopaminergic agonists (amphetamine, apomorphine, bromocriptine), are related only to the dopaminergic system [22, 36]. Several studies suggest that the activation of mesolimbic dopamine neurons arising from the cell bodies of the ventral tegmental area (VTA) and projecting to the nucleus accumbens plays an important role in mediating the suppression of tonic pain [37].

In order to further investigate the action of *T. catigua* extract on the dopaminergic system, we evaluated its effect on the hypothermia induced by apomorphine and also on haloperidol-induced catalepsy. It has been suggested that apomorphine-induced hypothermia results from two effects [38]: the first, observed at small doses (until 2 mg kg^−1^) are antagonized by neuroleptics; the second, induced by high doses (over 10 mg kg^−1^) are antagonized by antidepressants. In this study, the extract potentiated apomorphine-induced hypothermia, an action that could indicate an agonistic effect on the dopaminergic system. This result was further confirmed by using the catalepsy test, where the extract was able to prevent the cataleptic effect of haloperidol in a dose-dependent manner. The fact that *T. catigua* extract has stimulating effects upon the dopaminergic system is in agreement with previous *in vitro* results from our laboratory. In this context, Campos et al. [8] found a significant inhibition of dopamine uptake (IC_50_ = 35 *μ*g ml^−1^), and an increase in dopamine release (EC_50_ = 23 *μ*g ml^−1^) by *T. catigua* extract. It is important to remark that this is not an amphetamine-like effect, since the extract doses of 200 and 400 mg kg^−1^ (i.p.) did not significantly affect locomotor activity in mice [8].

Finally, aiming to identify the substance that could be responsible for the activity of *T. catigua* extract, we also tested some purified substances, named Fraction 2, Compound 2 and cinchonain IIB. However, none of them showed antinociceptive effects when assessed in the hot-plate test (data not shown). This result, together with the observation that in the hot-plate the effect of the extract are significant only 3 h after the treatment, lasting for up to 6 h, led us to hypothesize that the active substances from *T. catigua* extract could be related with some metabolites present in the plasma. Some analgesic substances, such as tramadol, morphine and tricyclic antidepressants [39–41], also have active metabolites. Additional experiments are necessary to confirm this hypothesis.

In summary, many authors have already shown that depression and pain are interconnected disorders, both pathophysiologically and therapeutically [42–44]. In this context, *T. catigua* hydroalcoholic extract presented relevant effects on the CNS, suggesting that the extract and/or active principles might represent potential therapeutic options for the treatment of depression and pain-related diseases, by a mechanism of action likely to be different from that of the clinically available drugs. Consequently, further experiments are now in progress to investigate and to identify the active substance(s) involved in these effects.

## Funding

Conselho Nacional de Desenvolvimento Científico e Tecnológico (CNPq), the Coordenação de Aperfeiçoamento de Pessoal de Nível Superior (CAPES), the Fundação de Apoio a Pesquisa do Estado de Santa Catarina (FAPESC), Financiadora de Estudos e Projetos (FINEP) and Laboratórios Catarinense, all from Brazil. A. F. V. and E. M. M. hold postdoctoral fellowships from CNPq.

## Figures and Tables

**Table 1:** Influence of several drugs on *T. catigua* hydroalcoholic extract antinociception effect expressed as MPE (%).

Treatment (dose)	Antagonist (dose)	Site/mechanism of action	MPE (%)
Morphine (5 mg kg^−1^, s.c.)	PBS	—	100
	Naloxone (2.5 mg kg^−1^, i.p.)	Non-specific opioid receptor antagonist	0
*T. catigua* (200 mg kg^−1^, p.o.)	PBS	—	39 ± 10
	Prazosin (1 mg kg^−1^, i.p.)	*α*_1_-adrenergic receptor antagonist,	38 ± 8
	SR141716A (10 mg kg^−1^, i.p.)	Cannabinoid receptor antagonist	45 ± 7
	SCH23390 (15 *μ*g kg^−1^, i.p.)	D_1_ receptor antagonist	0
	Sulpiride (50 mg kg^−1^, i.p.)	D_2_ receptor antagonist	48 ± 12
	Bicuculline (1 mg kg^−1^, i.p.)	GABA_A_ receptor antagonist	22 ± 10
	Naloxone (2.5 mg kg^−1^, i.p.)	Non-specific opioid receptor antagonist	20 ± 13
	PCPA (100 mg kg^−1^, i.p.)	Serotonin synthesis inhibitor	48 ± 8

Each group represents the mean  ±  SEM of 10 animals.

**Table 2:** Effect of *T. catigua* extract on Haloperidol (1 mg kg^−1^, i.p.)-induced catalepsy

Treatments	Catalepsy time (s)
PBS + PBS	1.8 ± 0.8
PBS + Haloperidol	58.4 ± 13.75***
PBS + *T. catigua* (200 mg kg^−1^)	2.0 ± 0.6
PBS + *T. catigua* (400 mg kg^−1^)	2.2 ± 0.6
*T. catigua* (200 mg kg^−1^) + Haloperidol	31.4 ± 2.4^∗,b^
*T. catigua* (400 mg kg^−1^) + Haloperidol	13.3 ± 3.7^c,$^

Each group represents the mean  ±  SEM of 10 animals.

***P < .001, *P < .05 compared with PBS; ^b^ *P* < .001 compared with Haloperidol; ^$^ *P* < .001 compared with *T. catigua* (200 mg kg^−1^) + Haloperidol.
